# Miscellany

**Published:** 1888-03

**Authors:** 


### Miscellany.

Diphtheria.—Dr. Johnson says: I have prescribed, for diphtheria, dilute acetic acid, about one part in ten, or as strong as can be agreeably borne, either as a gargle or with atomizer, and in every instance it has dissolved the exudate so rapidly that it was expectorated without much trouble. I direct that it be used for a period of five minutes, or as long as it can be done without greatly fatiguing the patient, at intervals of four or five hours, or often enough to keep the throat clear, and have no trouble in keeping the membrane from thickening and causing distress by its presence. Another thing: they all say that their throats feel easier and not so dry after using it. Since then I have treated about thirty cases, and, using acetic acid in some form, all have recovered quickly.

The *American Lancet,* commenting upon the despairing appeal, published in our columns, in behalf of the Buffalo Medical Library Association, very truly says: "Without any positive knowledge of the facts, we venture the opinion that the trouble is not that the physicians do not want a medical library, or that they are unable or unwilling to support one, but because there has not appeared in their midst a leader in this direction. If they find some man like Dr. Jas. R. Chadwick, of Boston, who loved books and has perseverance and tact, he would raise for the library in Buffalo thousands of dollars yearly, and thousands of volumes. Medical libraries do not grow of themselves. Lots of self-sacrificing work is needful in order that they may be established."

A Chinese Remedy for Croup.—The formula of Tcheng-Ke-Tang is as follows: "Take from an old well seven big spider-webs, two of which at least should contain living spiders. Mash them up together (the webs and spiders), and add to the mass (or *mess,* we should say,) about two scruples of alum dissolved in as little water as possible. Cook the whole over the fire until dry, and then increase the heat to incineration. The incinerated residue is powdered very fine, and the powder is blown into the throat with a little bamboo cane. This is a sure cure." In short, the Chinese use burnt alum. —*St. Louis Medical and Surgical Journal.*

---

Pharmaceutical Substitution.—A short time ago, a German association set itself the task of *proving* that the German pharmacists habitually *substituted* one ingredient for another when not in stock. Bogus prescriptions were, therefore, prepared containing unknown and absurd articles. One prescription read:

Aconit. Nap.
Tuber Cinereum.

Notwithstanding the fact that "Tuber Cinereum" is the "*tubercle of Gray substance at the base of the human brain*," this article with aconite was actually dispensed and paid for in *fifth-eight* Berlin pharmacies. Other fancy ingredients, such as *Urticaria Rubra*, *pemphygus foliaceus*, etc., etc., were dispensed in *seventy-seven* pharmacies; only twelve refused them. When the hoax was complete, the whole bundle of prescriptions, together with the compounds dispensed, were submitted to the editors of the journals. The organs of German pharmacy admit the truth of the charge. The Berlin Pharmaceutical Society, at their last meeting, *discreetly* resolved to take no notice of the matter. *The boasted superiority of German pharmacists would seem to have received a shock by these revelations.*

---

Tape-Worm.—The most successful way to get rid of him is by making him let go with his hooks. You must give him a narcotic remedy. We have one remedy that is the best for the armed worm, "tænia solium." Pomegranate, I do not believe, will ever fail, if properly applied. First, clear out the canal. A purgative will not do this. Give remedies that liquefy, such as phosphate of soda, for a few days; then, an active purge. The sodium phosphate must be given in the intervals of digestion, in decided doses. Then give:

℞—Pomegranate . . . . . . . . . . . . . . . . oz. iv.
Aq. font. . . . . . . . . . . . . . . . . . . . Oij.
Boil down to Oj, and give largely. —*Bartholow.*

The thirty-ninth annual session of the American Medical Association will be held in Cincinnati, O., on Tuesday, Wednesday, Thursday and Friday, May 8th, 9th, 10th and 11th, commencing on Tuesday, at 11 A. M. Members desiring to present papers should forward the paper or its title to W. W. Dawson, M. D., Cincinnati, O., Chairman Committee of Arrangements, at least one month before the meeting.

---

The past few months have witnessed the deaths of many prominent American medical men, among whom may be mentioned Dr. Asa Gray, the eminent botanist; Dr. Moses Gunn, Professor of Pathology, Rush Medical College; Dr. Alonzo Palmer, Professor of Medicine at Ann Arbor, and Dr. Wesley Carpenter, of New York.

---

The University of Pennsylvania gives a course to its medical students on "Medical Dietetics." Nothing can be more important, and the wonder is that medical colleges have neglected it so long.

---

Dr. Fordyce Barker says that the most valuable remedy for hemorrhages occurring near or at the climacteric is a combination of equal parts of fluid extracts of hamemelis and hydrastis.

---

The *Dietetic Gazette*, formerly the journal of Dietetics, appears in a new and enlarged form. This journal fills one of the few places hitherto vacant in medical periodical literature.

---

A single vesication over the fourth and fifth dorsal vertebræ puts an end at once to the sickness of pregnancy for the whole remaining period of gestation.

---

Sir Andrew Clark, of London, said: "I worked twelve years for bread, twelve for butter, and twelve more for the luxuries of life."

---

A number of Italian apothecaries, who deserted their posts during the recent cholera epidemic at Naples, have been fined fifty lire each.

OUR grandfathers used to cure the headache by kissing a pretty girl. Some of these old-time remedies are difficult to improve on.

---

Dr. Andur recommends rescrcin (gr. x-xx) for the relief of sea-sickness.

---

MEASLES is unusually prevalent in Buffalo at present. There are, however, but few fatal cases.

---

THE French Canadian physicians of New England have organized a medical association.

---

BERTI, of Italy, reports a case of organic impotence due to absence of the dorsalis penis artery.

